# Introduction to *Python Dynamic Diffraction Toolkit* (*PyDDT*): structural refinement of single crystals via X-ray phase measurements

**DOI:** 10.1107/S1600576723005800

**Published:** 2023-08-16

**Authors:** Rafaela F. S. Penacchio, Maurício B. Estradiote, Cláudio M. R. Remédios, Guilherme A. Calligaris, Milton S. Torikachvili, Stefan W. Kycia, Sérgio L. Morelhão

**Affiliations:** a Institute of Physics, University of São Paulo, São Paulo, SP, Brazil; b Faculdade de Física, Universidade Federal do Pará, Belém, PA, Brazil; c Brazilian Synchrotron Light Laboratory – SIRIUS/CNPEM, Campinas, SP, Brazil; d Department of Physics, San Diego State University, San Diego, California, USA; e Department of Physics, University of Guelph, Guelph, Ontario, Canada; HPSTAR and Harbin Institute of Technology, People’s Republic of China

**Keywords:** X-ray dynamic diffraction, single crystals, triplet phase determination, Python code

## Abstract

*PyDDT* is a free Python package of computer codes for exploiting X-ray dynamic multiple diffraction in single crystals. A wide range of tools are available for evaluating the usefulness of the method, planning feasible experiments, extracting phase information from experimental data and further improving model structures of known materials. Graphical tools are also useful in analytical methodologies related to the three-dimensional aspect of multiple diffraction. For general X-ray users, the *PyDDT* tutorials provide the insight needed to understand the principles of phase measurements and other related methodologies. Key points behind structure refinement using the current approach are presented, and the main features of *PyDDT* are illustrated for amino acid and filled skutterudite single crystals.

## Abstract 2

Computer simulation tools are presented for the easy exploration of dynamic diffraction in refining structural details of known materials. Here several practical examples illustrate their usage, covering all steps from designing of experiments to data analysis.

## 1. Introduction

Multiple diffraction (MD) peaks exhibiting asymmetrical line profiles in azimuthal scans of weak Bragg reflections are direct evidence of dynamical diffraction within perfect crystalline domains (Renninger, 1937 ▸; Chang, 1984 ▸). As the profile asymmetries are related to the structure factor phases of two or more simultaneously excited *hkl* reflections, MD experiments have been used as a refinement tool capable of uncovering structural details beyond the resolution limit of other techniques. A list of applications mainly related to phase measurements includes improvement of diffraction anomalous fine structure techniques (Lee *et al.*, 2006 ▸; Liu *et al.*, 2016 ▸), studies of orbital and charge ordering in magnetic materials (Shen *et al.*, 2006 ▸; Weng *et al.*, 2012 ▸; Liu *et al.*, 2017 ▸), probing minor internal strain induced by ion doping in optical crystals (Morelhão *et al.*, 2011 ▸; Amirkhanyan *et al.*, 2014 ▸), identification of chirality in crystals with no resonant atoms (Hümmer & Weckert, 1995 ▸; Shen *et al.*, 2000 ▸; Morelhão *et al.*, 2015 ▸; Kozlovskaya *et al.*, 2021 ▸), validation of ionic model structures for amino acid crystals (Morelhão *et al.*, 2017 ▸) and inspection of phonon scattering mechanisms in thermoelectric materials (Valério *et al.*, 2020 ▸). This list of successful applications has been possible after decades of knowledge in theoretical approaches and experimental procedures to process MD line-profile asymmetries into structural information (Hart & Lang, 1961 ▸; Colella, 1974 ▸; Post, 1977 ▸; Chapman *et al.*, 1981 ▸; Juretschke, 1982 ▸; Chang, 1997 ▸; Weckert & Hümmer, 1997 ▸; Chang *et al.*, 1999 ▸; Wang *et al.*, 2001 ▸; Mo *et al.*, 2002 ▸; Morelhão & Kycia, 2002 ▸; Morelhão, 2003*b* ▸; Soares *et al.*, 2003 ▸; Shen, 2003 ▸; Thorkildsen *et al.*, 2003 ▸).

Besides phase measurements, MD-based optical devices for X-rays are applications under consideration (Souvorov *et al.*, 2004 ▸; Huang *et al.*, 2013 ▸, 2014 ▸; Tang *et al.*, 2021 ▸). At the same time, the intrinsic three-dimensional nature of MD events has opened up an entirely new branch of methodologies for analyzing bulk crystals and nanostructured devices: for instance, to detect subtle growth-induced lattice changes at the p.p.m. level of doped crystals for habit modification studies (Remédios *et al.*, 2008 ▸, 2010 ▸; Lan *et al.*, 2018 ▸), lattice constants, phase transitions, piezoelectric and thermal expansion coefficients of single crystals (Avanci *et al.*, 1998*a* ▸; Borcha *et al.*, 2009 ▸; Tsai *et al.*, 2011 ▸; dos Santos *et al.*, 2019 ▸), anisotropic strain on ion-beam-induced crystallization of nanoparticles (Lang *et al.*, 2012 ▸, 2013 ▸), interface lattice strain (Sun *et al.*, 2006 ▸, 2007 ▸; Zheng *et al.*, 2016 ▸), spatial orientation of crystal domains in piezoelectrics (Morelhão *et al.*, 1999 ▸), thin films (Morelhão & Domagala, 2007 ▸) and nanostructures (Martínez-Tomás *et al.*, 2013 ▸), crystalline perfection of surfaces and epitaxial films (Morelhão & Cardoso, 1996 ▸; Hayashi *et al.*, 1997 ▸; Avanci *et al.*, 1998*b* ▸), and lattice mismatch of heteroepitaxial systems from semiconductors to topological insulators (de Menezes *et al.*, 2010 ▸; Domagała *et al.*, 2016 ▸; Smith *et al.*, 2017 ▸; Borcha *et al.*, 2017 ▸; Morelhão *et al.*, 2018 ▸, 2019 ▸). Diffuse multiple scattering is another MD-related phenomenon, observable on area detectors when using high-flux X-ray beams from advanced synchrotron facilities (Nisbet *et al.*, 2015 ▸). Although this phenomenon also carries detailed 3D information about the crystal structure, its predictability and practical applications are still open to investigation.

To explain and further exploit the potential of dynamic diffraction as a refinement tool, we introduce in this work the *Python Dynamic Diffraction Toolkit* (*PyDDT*), available on GitHub (Penacchio, 2022 ▸). It is a package of computer codes to guide general X-ray users through all the necessary steps to succeed in acquiring and analyzing dynamic MD data: understand the potential of dynamic diffraction in studying a material that can be grown as single crystals with millimetre-scale dimensions, select X-ray energy and measurable MD events intended to refine the average crystal unit cell, extract and index a list of useful profile asymmetries present on azimuthal scans, generate model structures, and compare model-predicted asymmetries with experimental ones. In addition, available graphical tools can help users in dealing with the 3D aspect of MD, such as a quantitative geometric description of MD peak splitting due to anisotropic lattice strain, the 2D intensity distribution near MD events, and directional prediction of multiple diffuse scattering contributions. To demonstrate the key concepts behind structure refinement via dynamic diffraction and the use of graphical tools, we start with one of the most traditional problems in X-ray crystallography: the contribution of chemical bonding charges to the diffracted intensities.

## 2. Covalent bonds in silicon crystals

**Figure 1:**
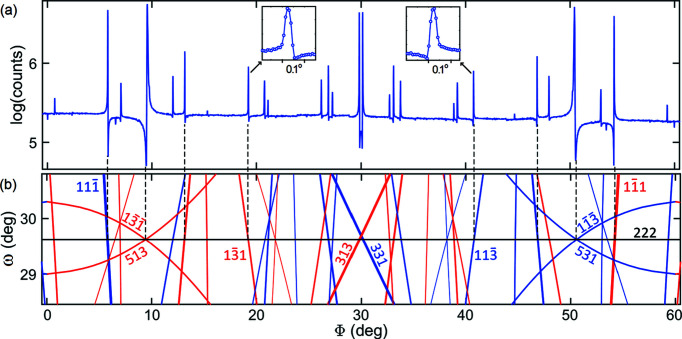
(*a*) A Φ scan of silicon reflection 222. Synchrotron X-rays of 8 keV, σ polarization. Peaks related by the sixfold symmetry of the [111] rotation axis have opposite line profile asymmetries, like the highlighted peaks (insets). (*b*) Graphical indexing of MD peaks by Bragg cone lines, where MD events are excited at the intersection of BC lines. The horizontal solid black line at ω = 29.76° denotes the BC line of reflection 222, while the red/blue lines indicate secondary reflections in the diffraction geometries *g* = 1 (red) and *g* = −1 (blue). The sense of sample rotation is clockwise with the 222 diffraction vector pointing to the observer.

Since the work of Ewald & Hönl (1936 ▸), the non-vanishing intensity of the silicon 222 reflection has been assigned to charge density along the covalent bonds. Later studies have shown that anharmonic effects due to noncentrosymmetric atomic sites also contribute to the 222 intensity above room temperature more than any other structural imperfections (Colella & Merlini, 1966 ▸; Roberto *et al.*, 1974 ▸; Colella, 1977 ▸). As the experimental evidence of bonding and anharmonic contributions has come from intensity measurements of extremely weak reflections, the occurrence of MD was carefully avoided. However, there is something curious about the MD events having 222 as the reference reflection: all triplet phases Ψ are close to either 0 or 180° in model structures where electronic charges are distributed along the bonds. Therefore, azimuthal Φ scans of the 222 reflection, as presented in Fig. 1 ▸(*a*), provide one of the best showcases of how the MD excitation geometry *g* = ±1 and the triplet phase Ψ are unambiguously correlated to the observable line profile asymmetry of any isolated MD peak. This correlation is written as (Morelhão *et al.*, 2017 ▸; Valério *et al.*, 2020 ▸)

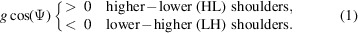

For instance, the MD peaks with 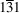 and 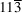 secondary reflections have the same phase value of Ψ = 5.4°, but they are excited under different geometries, *g* = 1 and *g* = −1, respectively. This results in MD peaks with opposite line-profile asymmetries, as seen in the insets of Fig. 1 ▸(*a*). The MD peak at Φ ≃ 19° has its left shoulder higher (H) than the right shoulder, which is lower (L), hence the labeled HL (higher–lower shoulders) type of asymmetry. The LH (lower–higher shoulders) type of asymmetry is observed for the structurally equivalent MD peak at Φ ≃ 41°, where only the geometry of excitation has changed from *g* = 1 to *g* = −1, as indicated in Fig. 1 ▸(*b*) by Bragg cone (BC) lines of different colors.

A BC line is a 2D graphic representation of Bragg’s law as a function of the instrumental angles ω and Φ, as detailed elsewhere (Domagała *et al.*, 2016 ▸). During the Φ rotation, the reciprocal-lattice point of the secondary reflection crosses the scattering sphere, also known as Ewald’s sphere, twice, first when moving from outside to inside the sphere, the *g* = −1 geometry, and second when moving from inside to outside the sphere, the *g* = 1 geometry. A clockwise crystal rotation around the Φ axis has been assumed as the positive sense. In Fig. 1 ▸(*b*), these excitation geometries are represented by the BC lines in blue (*g* = −1) and red (*g* = 1). In addition to indexing azimuthal scans, there are other MD-related phenomena where BC line plots can be helpful: revealing surface structure information via a specific MD geometry, otherwise known as Bragg surface diffraction (Jen & Chang, 1992 ▸; Morelhão & Cardoso, 1993 ▸; Hayashi *et al.*, 1997 ▸; Avanci & Morelhão, 2000 ▸; Avanci *et al.*, 1998*a* ▸; Freitas *et al.*, 2009 ▸; Lang *et al.*, 2013 ▸; Huang *et al.*, 2014 ▸; Zheng *et al.*, 2016 ▸), where the 2D intensity distribution around the MD peaks follows the orientation of the BC lines. When single crystals are hit with a high-flux (∼10^14^ ph s^−1^ mm^−2^) monochromatic X-ray beam, diffuse multiple scattering (Nisbet *et al.*, 2015 ▸) makes BC lines visible on zero-noise area detectors.

**Figure 2:**
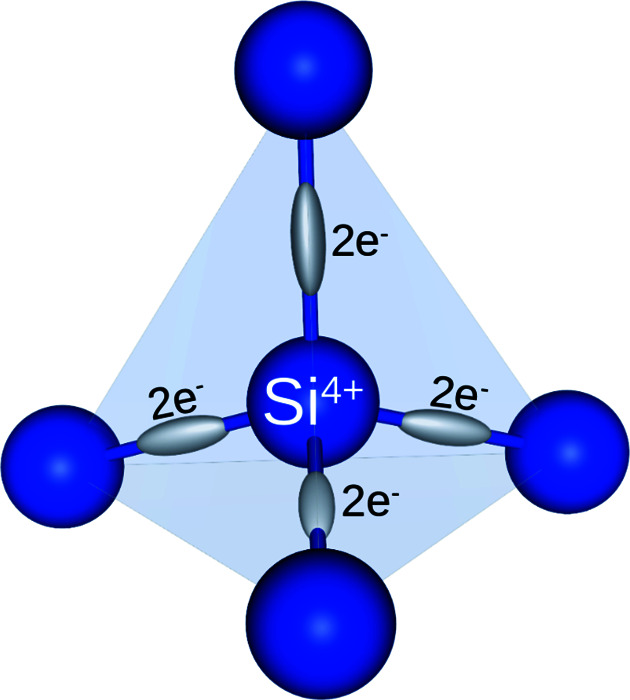
Model structure of a silicon crystal with electronic charges placed at the covalent bonds. Structure factors were computed using the atomic scattering factors of the Si^4+^ ion for the atomic sites and hydrogen for each electron in the bonds.

The simple rule summarized by equation (1) for correlating structural information and experimental results is the foundation upon which *PyDDT* was built. The structural information comes from the triplet phase values, calculated as in 

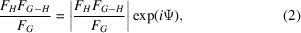

where *F*_*X*_ (*X* = *G*, *H* and *G*–*H*) stands for the structure factor of each involved reflection. *G* is the reference reflection, *i.e.* 222 in the above example, *H* is the secondary reflection excited during the Φ scan, and *G*–*H* is the coupling reflection responsible for the second-order rescattering process. The experimental results are the observed HL or LH asymmetries of the MD peaks. The asymmetries are the signature of a crystal undergoing dynamic diffraction, *i.e.* the multiple diffracted wavefields interfering with each other as they propagate inside the crystal (Weckert & Hümmer, 1997 ▸). The strategy of analyzing only the type of asymmetry, instead of measuring the triplet phase value itself from the line profile asymmetry, falls into the imponderability of knowing the lattice coherence length of each sample. In other words, lattice defects reduce the coherence of the diffracted wavefields, causing asymmetric profiles from the dynamic MD theory to appear as nearly symmetrical (Morelhão *et al.*, 2011 ▸). In this introductory example of structure modeling via dynamic diffraction, the presence of electronic charges in the covalent bonds, as schematized in Fig. 2 ▸, is necessary for a non-zero structure factor of the reference reflection, *i.e.* the Si 222 reflection. On top of this, the model also explains all observed peak asymmetries, implying that the 222 reflection phase itself, and hence the calculated triplet phases in Table 1 ▸, agree with the experimental data in Fig. 1 ▸(*a*).

In addition to isolated three-beam MD events where only one secondary reflection is excited, systematic four-beam MD events also obey the asymmetry rule in equation (1) and can be used for phasing purposes. They consist of two simultaneous three-beam MD peaks with identical triplet phases at any X-ray energy. For instance, for the MD peak at Φ ≃ 9.5° in Fig. 1 ▸(*a*), both the 513 and 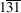 secondary reflections lead to Ψ = −174.5°. As both reflections have geometry *g* = 1 (red BC lines), the LH asymmetry is observed. On the other hand, simultaneous MD peaks with opposite geometries and identical Ψ values can also occur, leading to symmetrical MD peaks at mirroring Φ positions. The 313 and 331 MD peaks [Fig. 1 ▸(*a*)] are an example, as their combined profile is symmetric around Φ = 30°. At 8 keV these peaks are set apart by 0.34°, and hence they are not systematic MD peaks but rather nearly coincidental, requiring an energy increment of 31 eV to become a single peak with a perfectly symmetrical line profile. The silicon 222 reflection has a sixfold symmetry axis, implying a Φ scan that repeats itself every 60°, giving rise to 12 mirroring positions: six with double peaks such as those at Φ = 30°, and six with a tiny intensity dip as seen at Φ = 0° and Φ = 60° in Fig. 1 ▸(*a*). Each of these dips in intensity denotes the excitation of the secondary reflection 220, whose coupling reflection 002 is prohibited, *F*_002_ = 0, even in the silicon model with bonding charges. MD peaks with dip-like line profiles are caused by the *Aufhellung* effect that generally occurs when |*F*_*G*_| ≫ |*F*_*H*_ *F*_*G*–*H*_| (Chang, 1984 ▸; Morelhão *et al.*, 2005*b* ▸).

Structure refinement through *PyDDT* uses the entire data set of MD events whose line profiles are dominated by the second-order term of the *n*-beam dynamic theory of X-ray diffraction (Colella, 1974 ▸; Chang, 1984 ▸; Weckert & Hümmer, 1997 ▸; Morelhão, 2003*a* ▸). These are the profiles that obey the asymmetry rule in equation (1). The best approach to ensure the validity of this rule for most *H* reflections is by selecting weak reference reflections such that |*F*_*G*_| ≪ |*F*_*H*_ *F*_*G*–*H*_|. For instance, according to the model structure in Fig. 2 ▸, the Si 222 reference reflection has |*F*_*G*_| = 6.5, while the weakest MD peak listed in Table 1 ▸ has *H* as the 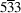 reflection and |*F*_*H*_ *F*_*G*–*H*_| = 638. As a visual criterion, nearly symmetric MD peaks, where the asymmetries are limited to very low intensity shoulders, generally follow the rule in equation (1). Exceptions are related to the overlap of MD peaks when it compromises the identification of their individual asymmetries. Plotting BC lines, as in Fig. 1 ▸(*b*), is helpful to visualize such overlap events even when they appear as a single MD peak. Another exception is caused by polarization suppression of the second-order term, an unusual situation that can occur when the *H* or *G*–*H* reflections have a Bragg angle close to 45°, as discussed extensively elsewhere (Stetsko *et al.*, 2000 ▸; Morelhão & Avanci, 2001 ▸; Morelhão *et al.*, 2005*a* ▸,*c* ▸).

## 3. Capabilities of *PyDDT*

Usage of *PyDDT* can be divided into a few main procedures as follows.

(i) To understand the potential of dynamic diffraction in the structure refinement of single crystals, *PyDDT* allows users to compare the structure factor phases of different models with subtle changes in ionic charges, Debye–Waller factors, occupation factors, bonding charges, internal lattice strain and any other structural modification describable through an average crystal unit cell. On the basis of the list of susceptible reflection phases, measurable reflections are selected as the reference reflection *G*, and the triplet phases are computed for the proposed model structures. Graphical output showing all MD events with the flipping of profile asymmetries between models provides a clear perspective of how helpful dynamic diffraction via azimuthal scans can be in terms of finding improved model structures of the materials. See Section 3.1 for a few examples.

(ii) Selection of the X-ray energy to enhance phase variation between models is another capability of *PyDDT*. It also allows the identification of resonant phase shifts, a handy tool for planning multiwavelength anomalous diffraction experiments, where the MD asymmetries are scanned against the reference reflection phase as it shifts as a function of X-ray energy. For each wavelength/energy, completely different data sets of profile asymmetries are obtained, which can significantly narrow the range of feasible model structures. A demonstration of this phenomenon is given in Section 3.2.

(iii) With an azimuthal scan in hand, 2D graphs of the BC lines, *e.g.* Fig. 1 ▸(*b*), are available via *PyDDT* to help with azimuth offset correction, identification of MD excitation geometry *g* and discarding of overlapped peaks whose asymmetries may behave differently than predicted by equation (1). After the offset correction, the MD peak positions are determined and their secondary reflections *H* indexed. Line profile fitting of each MD peak is performed automatically to determine the degrees of asymmetry and reliability, as described in Section 3.3. By the end of this procedure, there is a list of *H* reflection indices with their *g* = −1 or 1 values and observed HL or LH type of line-profile asymmetries.

(iv) An asymmetry comparison diagram (ACD) is the final output of *PyDDT*, given to the user as a graphical tool to organize and compare the whole set of observed asymmetries against the model-predicted ones. A few examples of this *PyDDT* capability are given in Section 3.4.

### 3.1. Finding suitable MD peaks

**Figure 3:**
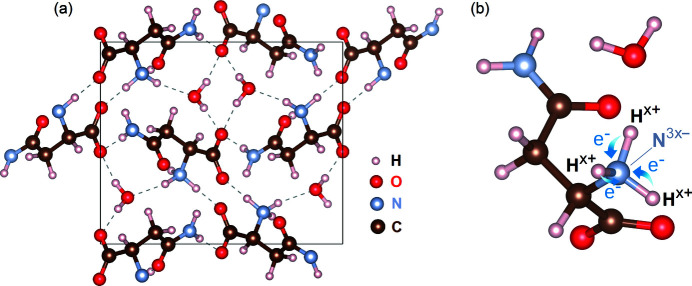
The crystal structure of l-asparagine monohydrate as determined from neutron diffraction (Verbist *et al.*, 1972 ▸). (*a*) The unit cell along the *a* axis, showing hydrogen bonds (dashed lines) between molecules. (*b*) A single asparagine molecule in the ionic model as a function of charge transferred *x* in electron number from hydrogen to nitrogen in the amino group.

From the crystallographic information file (CIF) of a compound, available built-in tools allow users to generate alternative model structures. Each model is a formatted input text file containing the chemical species and their atomic fractional coordinates, occupation factors and isotropic *B* factors, as described in the walkthrough of the *PyDDT* user’s guide (Penacchio, 2022 ▸). For a practical example, validation of ionic models is necessary for crystal structures determined by neutron diffraction, as demonstrated earlier when resolving the ionic model of a simple amino acid crystal, alanine, by carrying out a single Φ scan (Morelhão *et al.*, 2017 ▸). In the case of l-asparagine monohydrate (ASN), a more complex amino acid with water molecules in its crystal structure, different *B* factors for each atomic site can blur the resolution of electronic charge in the hydrogen bonds between the molecules [Fig. 3 ▸(*a*)], at least from the perspective of dynamic diffraction.

The interplay between ionic models and displacement parameters can be demonstrated by searching for reflection phases susceptible to the number of electrons transferred from hydrogen to other atoms, for instance between hydrogen and nitrogen in the amino group, as depicted in Fig. 3 ▸(*b*). For a partial charge transfer *x* in electron number, the corresponding model has an occupation factor 1 − *x* for each hydrogen in this group, while both the neutral nitrogen and the N^3−^ ion occupy the same site with occupancy factors 1 − *x* and *x*, respectively. For *x* = 0, all atoms are neutral, and there is one e^−^ at each hydrogen site. For *x* = 1, there are only non-X-ray scattering protons at the hydrogen sites, and the amino group has a spherically symmetric electronic charge distribution. In such models, the effective scattering factors of the H^*x*+^ and N^3*x*−^ ions in the amino group are 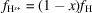 and 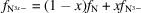 . *PyDDT* has a user-friendly interface for generating such model structures, including the Cromer–Mann coefficients to calculate the scattering factor *f* of atoms and ions (Brown *et al.*, 2006 ▸). Resonance amplitudes *f*′ + *if*′′, often called dispersion correction terms or anomalous scattering factors, are also accounted for in *f* via linear interpolation of the tabulated values for free atoms (Brown *et al.*, 2006 ▸; Morelhão, 2016 ▸).

**Figure 4:**
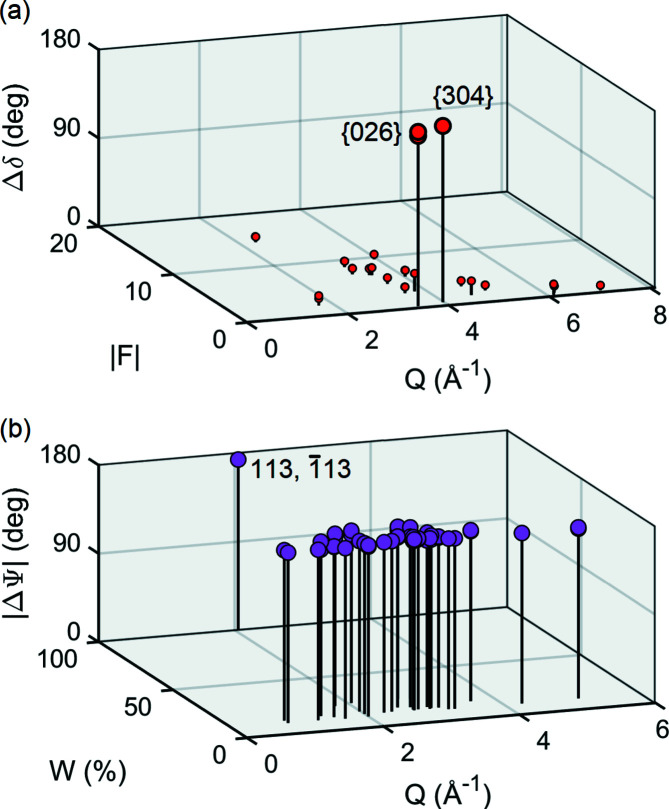
(*a*) The phase shift Δδ of structure factors *F* = |*F*|exp(*i*δ) regarding two ASN model structures. *Q* is the Bragg reflection diffraction-vector modulus. Only reflections with Δδ > 5° (vertical pins) are shown. (*b*) Predicted MD events with line-profile asymmetry flipping, *i.e.* where cos(Ψ)cos(Ψ + ΔΨ) < 0 (vertical pins), in the Φ scan of the reference reflection *G* = 026. *W* is the relative value of |*F*_*H*_ *F*_*G*–*H*_| and *Q* refers to the secondary reflections *H*. ΔΨ is the triplet phase shift between the two model structures.

Fig. 4 ▸(*a*) compares single reflection phases between two models, one for *x* = 0 and another for *x* = 1; both models have a mean *B* factor 〈*B*〉 = 6 Å^2^ for all atomic sites – the effect of different *B* factors for each atomic site is evaluated later in Section 3.4. As can be seen, two families of reflections, {026} and {304}, are highly susceptible to this particular charge distribution in the amino group. These reflections are also very weak, making them good candidates for reference reflections where the asymmetry rule in equation (1) is valid. A near 180° phase shift of the reference reflection implies a significant number of MD profiles having opposite asymmetries in the two models, as shown in Fig. 4 ▸(*b*) for 026 as the reference reflection. The Φ scan of the 026 reflection has the advantage that the strongest MD event, with 113 (or 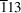 ) secondary reflection, is in principle able to discriminate between the two models.

### 3.2. Selection of X-ray energy

**Figure 5:**
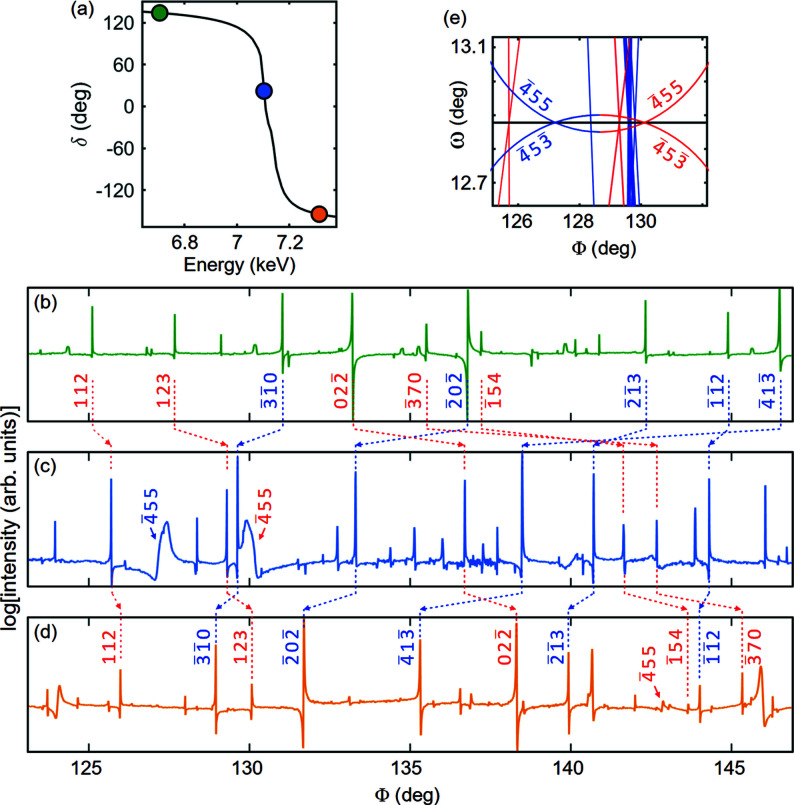
The resonant phase shift in SKD LaFe_4_P_12_. (*a*) The structure factor phase δ of reflection 002 as a function of X-ray energy (solid line). Cubic unit cell of edge 7.8316 Å with origin at the La^3+^ ions (Jeitschko & Braun, 1977 ▸). Colored circles are placed at the X-ray energies of the experimental data (see bottom panels). (*b*)–(*d*) Multiwavelength SKD 002 Φ scans collected on the Low-Energy Wiggler beamline of the Canadian Light Source (Leontowich *et al.*, 2021 ▸): (*b*) 6.705 keV, (*c*) 7.103 keV and (*d*) 7.314 keV. The reference for Φ = 0 is the [100] direction pointing upstream. MD peak position tracking between scans is indicated (dashed lines and arrows), as well as a few *hkl* indices of secondary reflections. As the energy increases, peaks with excitation geometries *g* = −1 (blue indices) and *g* = 1 (red indices) move in opposite senses: negative and positive, respectively. From the top (*b*) to the bottom (*d*) panel, peak asymmetries are observed to flip twice: HL → LH → HL or LH → HL → LH. (*e*) BC lines at 7.103 keV, slightly above the minimum energy for exciting MD 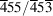 .

In materials containing elements whose absorption edges are in the spectral range of the radiation source, X-ray energies can be selected to enlarge the dynamic diffraction data set via the resonant phase shift. This recalls the multiwavelength anomalous diffraction approach used in protein crystallography, but with the difference that the experimental data monitored as a function of the X-ray energy are the MD peak asymmetries instead of diffracted intensities of individual Bragg reflections. For example, consider skutterudite LaFe_4_P_12_ (SKD) (Takegahara & Harima, 2007 ▸), whose 002 reflection undergoes a huge phase shift of about 280° across the Fe absorption edge [Fig. 5 ▸(*a*)]. Consequently, SKD 002 Φ scans have peak asymmetries extremely susceptible to X-ray energies close to 7.1 keV, such as those in Figs. 5 ▸(*b*)–5 ▸(*d*), where the peak asymmetries are observed to flip twice. For instance, MD 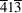 has Ψ = −108.8, 0.5 and 175.7° at the used energies of 6.705, 7.103 and 7.314 keV, respectively. Hence its HL asymmetry in Fig. 5 ▸(*b*) flips to LH in Fig. 5 ▸(*c*) and flips back to HL in Fig. 5 ▸(*d*). As the double flipping is mainly induced by the resonant phase shift of the reference reflection, it is observed for all MD peaks that were tracked from one scan to the other. In tracking peak positions as X-ray energy increases, the general rule is that MD events excited under the *g* = −1 (*g* = 1) geometry move in the negative (positive) sense of the Φ values. In other words, all MD events with blue *hkl* indices (*g* = −1) in Fig. 5 ▸(*b*) go to the left in Figs. 5 ▸(*c*) and 5 ▸(*d*), while the MD events with red indices (*g* = 1) go to the right. Model structures capable of explaining multiwavelength data sets of profile asymmetries in SKD are related to differences in the Debye–Waller factors for each atomic site, as pointed out elsewhere (Valério *et al.*, 2020 ▸; Penacchio, 2022 ▸).

For light materials such as amino acid crystals without metal ions, it is necessary to consider the benefits of using shorter or longer wavelengths according to the number of accessible MD peaks with a minimum of overlapping. This is another benefit of having graphical tools available to visualize BC lines and understand their behavior as a function of X-ray energy. Slightly above the minimum energy for exciting a given MD event, its line profile can become very broad as both diffraction geometries occur close to each other and can overlap many other MD events, *e.g.* the case reported in Fig. 5 ▸(*e*). A small increment in energy leads to drastic displacement in Φ, as illustrated by the 13° displacement of MD 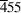 between Figs. 5 ▸(*c*) and 5 ▸(*d*), against an energy increment of only 211 eV. The usefulness of such cases is related more to the accurate determination of either lattice parameters or energy of synchrotron X-rays than to the asymmetry reading itself, and they can be better interpreted via graphical representation of the azimuthal scans as a function of X-ray wavelength (Rossmanith, 2003 ▸).

### 3.3. Indexing and asymmetry reading

**Figure 6:**
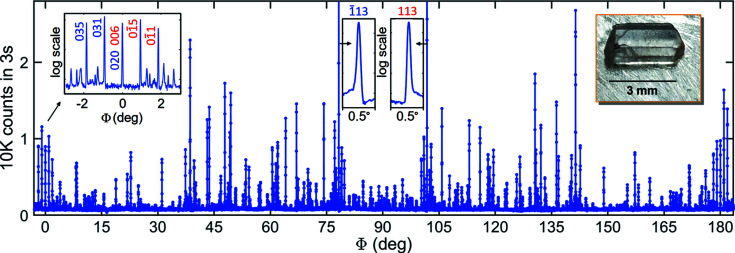
An ASN 026 Φ scan with Cu *K*α_1_ radiation. The mirroring position around Φ = 0, the strongest MD peaks 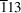 and 113, and a sample photograph are highlighted as insets. Secondary reflection indices in red (*g* = 1) and blue (*g* = −1) denote the diffraction geometry; see Section S1 for graphics of BC lines. The longest sample dimension corresponds to the *a* axis, the [100] lattice direction, taken as Φ = 0 when pointing upstream.

The larger the unit cell, the greater the number of MD peaks in the Φ scans. Indexing of organic crystals, in general with unit cells of volume above 500 Å^3^, can be challenging. The BC line graphs from *PyDDT* are very helpful, mainly for reference reflections along axes of low symmetry such as the alanine 261 reflection, a single-fold symmetry axis with no mirroring positions in the Φ scan (Morelhão *et al.*, 2017 ▸). The ASN 026 reflection is a twofold symmetry axis, making it much easier to identify the sample’s azimuth and therefore index the secondary reflections of the MD peaks. The 026 Φ scan repeats itself every 180°, giving rise to four mirror positions, three of which are seen in Fig. 6 ▸ at Φ = 0, 90 and 180°. The MD events with symmetric line profiles at either Φ = 0 or 180° are caused by the systematic simultaneous excitation of secondary reflections 020 and 006, taking place when the crystal’s [100] direction lies in the plane of incidence – the plane that contains the incident and 026 diffracted X-ray beams. The other secondary reflections were indexed assuming this direction points to the X-ray source at Φ = 0; see Section S1 in the supporting information for a graph of the BC lines. Once the sample azimuth is identified, *PyDDT* selects and indexes most peaks above the background noise. Nevertheless, the user needs to carry out a final check, deselecting overlapped peaks and including others that, for some reason, were skipped by the peak searching algorithm.

Several MD peaks in the ASN 026 Φ scan exhibit line profiles with readily distinguishable asymmetries, such as those highlighted in the insets of Fig. 6 ▸. This is the typical signature of dynamic diffraction and, therefore, of a sample of good crystalline quality. From only the asymmetries of a few MD peaks, it is already possible to rule out the model structure with neutral atoms, that is, the *x* = 0 model in Fig. 3 ▸(*b*). For instance, the MD peak at Φ ≃ 102° with secondary reflection 113 and geometry *g* = 1 exhibits the LH type of asymmetry, implying that it must have cos(Ψ) < 0 according to equation (1). The *x* = 0 and *x* = 1 models lead to triplet phases Ψ = −4.6° and Ψ = −177.9°, respectively. Equivalent results are obtained for the other indexed peaks in the insets of Fig. 6 ▸, all indicating that ionic models where *x* > 0 need to be evaluated. However, it is necessary to read as many asymmetries as possible for a more accurate evaluation of valid model structures.

**Figure 7:**
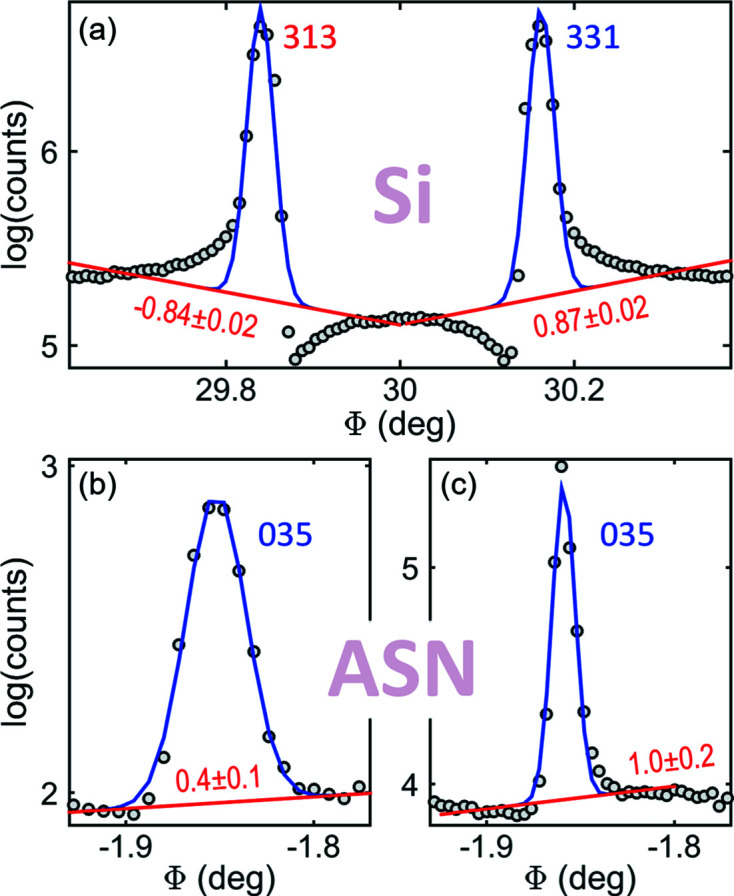
Asymmetry reading via line-profile fitting (blue solid lines) of the log-transformed data (scattered circles) using a Gaussian function with a sloping baseline (red solid lines). (*a*) Highly asymmetric MD peaks from an Si 222 Φ scan (Fig. 1 ▸). (*b*), (*c*) The smoothly asymmetric MD peak from ASN 026 Φ scans carried out with (*b*) characteristic and (*c*) synchrotron X-rays. Baseline slope values are indicated on each plot.

*PyDDT* comes with a line-profile fitting tool designed for asymmetry reading of isolated non-overlapped peaks. It consists of a Gaussian function with a sloping baseline to be adjusted to the log-transformed intensity data. Although the fit quality can be questionable for highly asymmetric peaks, as in Fig. 7 ▸(*a*), it is very efficient at identifying the peak asymmetry: a negative/positive slope indicates the HL/LH type of asymmetry. The slope and its uncertainty are useful for setting reliability criteria in the automatic asymmetry reading mode of *PyDDT*, a detail particularly relevant for line profiles with a low degree of asymmetry, such as those in Figs. 7 ▸(*b*) and 7 ▸(*c*).

**Figure 8:**
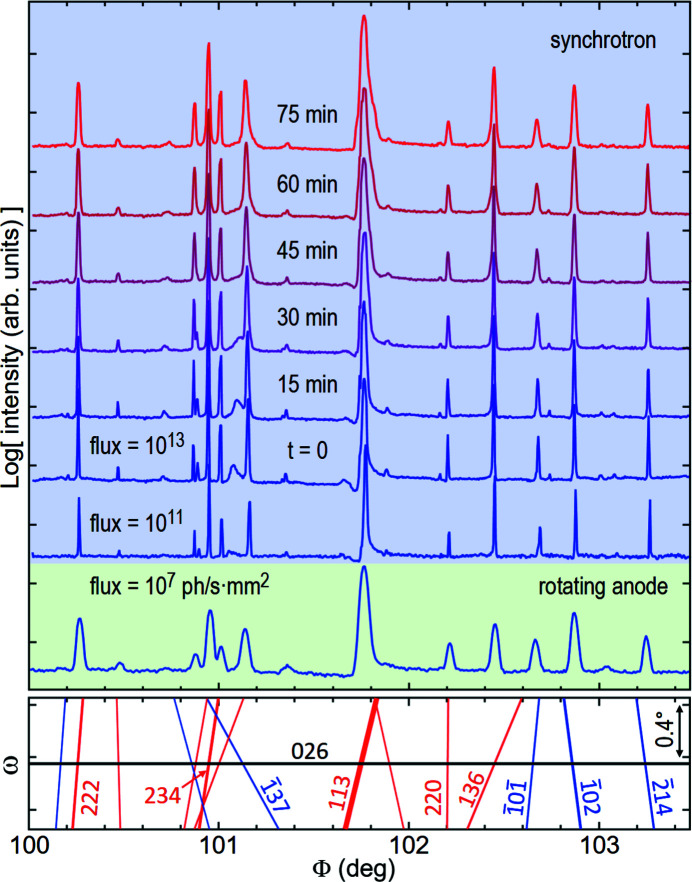
ASN 026 Φ scans collected in-house (rotating anode) and on the EMA beamline (synchrotron). For flux below 10^11^ ph s^−1^ mm^−2^, no changes in the MD line profiles were observed after 12 h of continuous exposure. A rapid increase in sample mosaicity and loss of sharpness of profile asymmetry occur for flux above 10^13^ ph s^−1^ mm^−2^, as can be seen from the first scan collected at time instant *t* = 0, when exposure to this high flux begins, to the last one collected 75 min later. (Bottom panel) A graphic of BC lines and indexed secondary reflections *H*.

The extent of the line-profile asymmetry is many times greater than the peak intrinsic width at half maximum. Instrumental broadening can smooth the asymmetry and reduce the MD signal-to-noise ratio, but the asymmetry reading is feasible with either characteristic or synchrotron radiation, provided that the axial divergence is limited to a few times the intrinsic width, in general below 1 mrad. Figs. 7 ▸(*b*) and 7 ▸(*c*) report slopes of 0.4 ± 0.1 and 1.0 ± 0.2, respectively, for the 035 MD peak of the ASN sample as measured on two different instruments; see Section S2 for more details about the fitting procedure. The former was measured with Cu *K*α_1_ radiation from a rotating-anode generator, whose conditioning beam optics and four-circle diffractometer have been described elsewhere (Morelhão *et al.*, 2018 ▸, 2019 ▸). The latter was measured on a high-flux low-divergence synchrotron beamline, the EMA beamline of the synchrotron light source SIRIUS, at the Brazilian Synchrotron Light Laboratory (LNLS) (dos Reis *et al.*, 2020 ▸). Low-divergence beams make the asymmetry sharper, reducing overlapping, but require scans with more data points. Concurrently, the combination of low divergence and narrow reference reflection can be very demanding in terms of instrumental stability to keep the baseline intensity constant during the Φ rotation. High-flux beams significantly reduce the counting time and, in general, improve the baseline statistical noise. However, in the case of a fragile crystal such as ASN, whose structure is stabilized by hydrogen bonds between the molecules, a flux above 10^13^ ph s^−1^ mm^−2^ hitting the sample at room temperature gives rise to the formation of hydrogen gas inside the crystals (Meents *et al.*, 2010 ▸). This quickly breaks the crystal from the inside out, creating mis­aligned domains and making the line profiles and the baseline more irregular. After extended exposure, the crystal becomes a mosaic of small domains no longer holding dynamic diffraction. The MD line profiles become broader with undetectable asymmetries. In Fig. 8 ▸, there is a comparison of the same Φ-scan session collected in-house and on the EMA beamline as a function of flux and time of exposure.

The overall effects of angular resolution and baseline instabilities explain the slope values with nearly the same relative uncertainties in both instruments, such as the values reported above [Figs. 7 ▸(*b*) and 7 ▸(*c*)], for which the relative uncertainties are 25 and 20%, respectively (see also Section S2 and the inset of Fig S3). The cut-off for the slope relative uncertainty is usually set to about 50%, *i.e.* only profile asymmetries of non-overlapping MD events with relative uncertainties smaller than 50% were considered for comparison with model-predicted asymmetries, as described in the next section.

### 3.4. Comparing structure models and experimental results

Model structures can depend on several variables. *PyDDT* has built-in tools to generate ACDs (asymmetry comparison diagrams) where experimental and model-predicted profile asymmetries are compared as a function of two variables per diagram (Valério *et al.*, 2020 ▸). For instance, one variable can be the charge transferred *x* in electron number from hydrogen to nitrogen in the amino group, as depicted in Fig. 3 ▸(*b*). The other variable can be the relative value of the *B* factor for each atomic site with respect to the overall mean value 〈*B*〉. To be more specific, for the *n*th atomic site *B*_*n*_(*z*) = (1 − *z*)〈*B*〉 + *zB*_0,*n*_, where *z* ∈ [0, 1] and *B*_0,*n*_ stand for the actual values reported in the literature, such as from neutron diffraction in the case of alanine and ASN crystals (Lehmann *et al.*, 1972 ▸; Verbist *et al.*, 1972 ▸).

**Figure 9:**
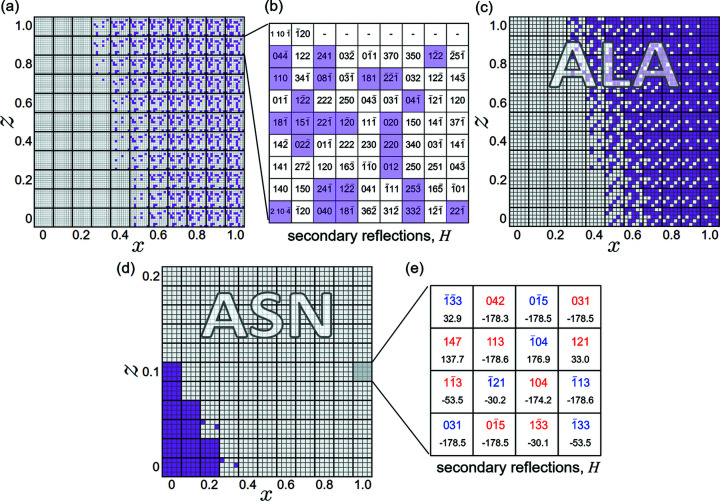
(*a*) ACD of alanine (ALA) as a function of the *x* and *z* model structure parameters. Purple-colored matrix positions indicate discrepant asymmetries. (*b*) Secondary *H* reflection indices for each matrix position; susceptible MD peaks are highlighted. (*c*) ACD considering the susceptible (highlighted) MD peaks only. (*d*) ACD of asparagine (ASN) based on susceptible MD peaks from the in-house 026 Φ scan (Fig. 6 ▸). (*e*) *H* reflection indices for each matrix position. Blue or red indices denote *g* = −1 or *g* = 1 diffraction geometry, respectively. Triplet phase Ψ values (in degrees) are shown below each index.

An ACD consists of a grid of square matrices, where each matrix represents a model structure and each position in a matrix represents one of the measured profile asymmetries, *i.e.* one MD event of the Φ scan. For viewing purposes, every matrix position is colored differently according to whether the corresponding profile asymmetry matches or differs from the model-predicted one. Fig. 9 ▸ shows examples of ACDs as a function of *x* and *z* variables for two amino acid crystals. For the ACD of alanine, the MD data set from the synchrotron 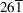 Φ scan is revisited (Morelhão *et al.*, 2017 ▸), this time via *PyDDT*, to assess the impact of *z* properly. For the ACD of l-asparagine monohydrate, the data set from its 026 Φ scan in Fig. 6 ▸ is used.

Initially, a preliminary ACD is constructed using the complete list of reliable profile asymmetries obtained from Φ scans, as in Fig. 9 ▸(*a*). It can be simplified by selecting only susceptible MD events, those highlighted in Fig. 9 ▸(*b*), whose corresponding matrix positions change in color as a function of the model parameters. The simplified ACD shown in Fig. 9 ▸(*c*) indicates an asymmetry data set much more susceptible to parameter *x* than parameter *z*. For models with different *B* factors for each atomic site at room temperature, *z* = 1, the experimental MD profile asymmetries agree with model structures where the amount of charge transferred from hydrogen to nitrogen in the amino group is limited to *x* ≤ 0.2. This limit increases to *x* ≤ 0.4 in the hypothetical case of a sample cooled to the point where *z* ≃ 0. In other words, the dynamic diffraction data set of alanine used here is detecting electronic charges at the hydrogen sites, implying compatible ionic models ranging from neutral atoms to one with H^0.2+^ and N^0.6−^ ions.

The situation is opposite with the ACD of asparagine in Fig. 9 ▸(*d*), constructed using the susceptible MD peaks indicated in Fig. 9 ▸(*e*), where the triplet phase values for a model with *x* = 1 and *z* = 0.1 are also displayed; see Section S3 for more details about the behavior of Ψ as a function of *x* and *z*.

Without accounting for the significantly different *B* factors, *z* < 0.1, only ionic models where *x* > 0.1 agree with the data set used. However, as different *B* factors are introduced into the models, *z* > 0.1, the predicted asymmetries become independent of *x* and they match the whole dynamic diffraction data set from the ASN 026 Φ scan. This is a significant result, demonstrating that all asymmetries with reliability better than 40% obtained via *PyDDT* perfectly match those predicted by models with the *B* factors from neutron diffraction at room temperature (Verbist *et al.*, 1972 ▸).

## 4. Final remarks

To exploit dynamic multiple diffraction as a structure refinement tool, it is imperative first to evaluate how susceptible the individual Bragg reflection phases are to structural variables within the broad spectrum of synchrotron X-rays. *PyDDT* is a package of computer codes to make such a preliminary evaluation as easy as possible. However, unlike deterministic methods in crystallography, phase measurements via azimuthal scans of Bragg reflections allow the narrowing of the range of feasible model structures of known materials. Therefore, before moving on to the experimental parts, from crystal synthesis to the actual realization of azimuthal scans of chosen reflections, potential users have to be as sure as possible that, in principle, the method is capable of elucidating new information about the material. Accessing electronic charges in covalent and hydrogen bonds were the main examples used in this work to illustrate the usage of *PyDDT*. It has been demonstrated that in amino acid crystals the atomic displacement parameters can affect the reflection phase susceptibility in detecting bond charges, a fact that has to be taken into account from the initial evaluation.

Resonant/anomalous phase shift is an asset with the potential for better resolution of displacement parameters, bonding charges and ionic models. Its occurrence can be easily identified via *PyDDT*, as demonstrated in predicting and measuring a remarkable phase shift in skutterudite LaFe_4_P_12_. Structure refinement of this class of thermoelectric materials by anomalous phase measurements is currently under investigation.

Orientation of single crystals with a multi-axis diffractometer can be a trivial task in many experiments. For azimuthal scan-based phase measurements there are two extra challenges. The sample has to be perfect enough to undergo dynamic multiple diffraction, and the azimuthal rotation demands mechanical stability to keep the reference reflection diffracting within the vertical beam divergence during the scans. On the instrumental side, modern synchrotron facilities with smaller beam sizes have improved sample stability and its sphere of confusion over the years, speeding up MD azimuthal scans. Without recourse to synchrotron X-rays, selecting and mounting good samples must be accomplished in supporting laboratories equipped with four-circle diffractometers and confocal optics, such as the in-house rotating-anode powered diffractometer used in this work.

## Supplementary Material

Additional analysis and figures. DOI: 10.1107/S1600576723005800/iu5039sup1.pdf

## Figures and Tables

**Table 1:** Secondary reflection *H* of MD events observed on the Si 222 Φ scan in Fig. 1 ▸(*a*), excitation geometry *g* = ±1 as defined in Fig. 1 ▸(*b*), triplet phases Ψ as calculated for a model structure with electronic charges placed at half the length of the chemical bonds (Fig. 2 ▸) and line-profile asymmetry (Asym.) according to equation (1)

*H*	Φ (°)	*g*	Ψ (°)	Asym.	*H*	Φ (°)	*g*	Ψ (°)	Asym.
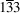	0.7	1	−174.6	LH	331	30.2	−1	4.1	LH
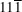	5.8	−1	−175.9	HL	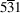	32.7	1	6.1	HL
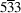	7.0	1	−173.4	LH	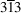	33.1	1	−175.1	LH
*513*	9.4	1	−174.5	LH	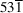	33.8	−1	5.5	LH
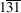	9.4	1	−174.5	LH	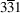	38.9	1	−174.6	LH
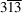	12.0	−1	5.5	LH	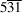	39.2	1	−173.4	LH
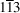	13.1	1	4.9	HL	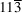	40.8	−1	5.4	LH
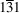	19.2	1	5.4	HL	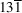	46.9	−1	4.9	LH
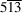	20.8	−1	−173.4	HL	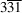	48.0	1	5.5	HL
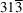	21.1	−1	−174.6	HL	531	50.6	−1	−174.5	HL
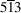	26.2	1	5.5	HL	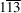	50.6	−1	−174.5	HL
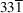	26.9	−1	−175.1	HL	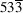	53.0	−1	−173.4	HL
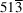	27.3	−1	6.1	LH	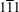	54.2	1	−175.9	LH
313	29.8	1	4.1	HL	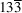	59.3	−1	−174.6	HL
