# Supplementary material for: Introduction to Python Dynamic Diffraction Toolkit (PyDDT): structural refinement of single crystals via X-ray phase measurements
Source: J Appl Crystallogr. 2023 Aug 16;56(Pt 5):1574–84. doi: 10.1107/S1600576723005800 (PMC10543681; doi:10.1107/S1600576723005800)
Supplement: Supplementary file 1 [file j-56-01574-sup1.pdf]

Support Information for article: Introduction to Python Dynamical Diffraction Toolkit (PyDDT): structural refinement of single crystals via X-ray phase measurements

Rafaela F. S. Penacchio,[a] Maurício B. Estradiote,[a] Cláudio M. R. Remédios,[b] Guilherme A. Calligaris,[c] Milton S. Torikachvili,[d] Stefan W. Kycia[e] and Sérgio L. Morelhão [a]*

[a]*Institute of Physics, University of São Paulo, São Paulo, SP, Brazil,* [b]*Faculdade de Física, Universidade Federal do Pará, Belém, PA, Brazil,* [c]*Brazilian Synchrotron Light Laboratory - SIRIUS/CNPEM, Campinas, SP, Brazil,* [d]*Department of Physics, San Diego State University, San Diego, CA, USA, and* [e]*Department of Physics, University of Guelph, Guelph, ON, Canada. E-mail: morelhao@if.usp.br*

S1. Indexing ASN 026 Φ-scan

The full Φ-scan of reflection 026 of L-asparagine monohydrate (ASN) presented in the main text was indexed by locating the systematic four-beam MD 020/006 that marks two identical mirroring positions set apart by 180°. It occurs with any X-ray energy as long as the 026 reflection fulfills the Bragg diffraction condition. This four-beam MD stands for two simultaneous three-beam MDs, one with secondary reflection $H = 020$ and the other with $H = 006$. Both MDs have exactly the same strength and triplet phase values, but opposite geometries (blue and red BC lines) that cancel out their individual asymmetries and give rise to a perfectly symmetric peak, as seen at $\Phi = 0$ in

Fig S1(a). There is also an intermediate position of mirroring at $\Phi = 90°$, Fig. S1(b), that appears as a consequence of the systematic ones at $\Phi = 0$ and 180°. Therefore, all phase information available in the ASN 026 Φ-scan can be acquired within a 90° interval, although wider scans are recommended to improve statistic and reliability of the asymmetry reading.

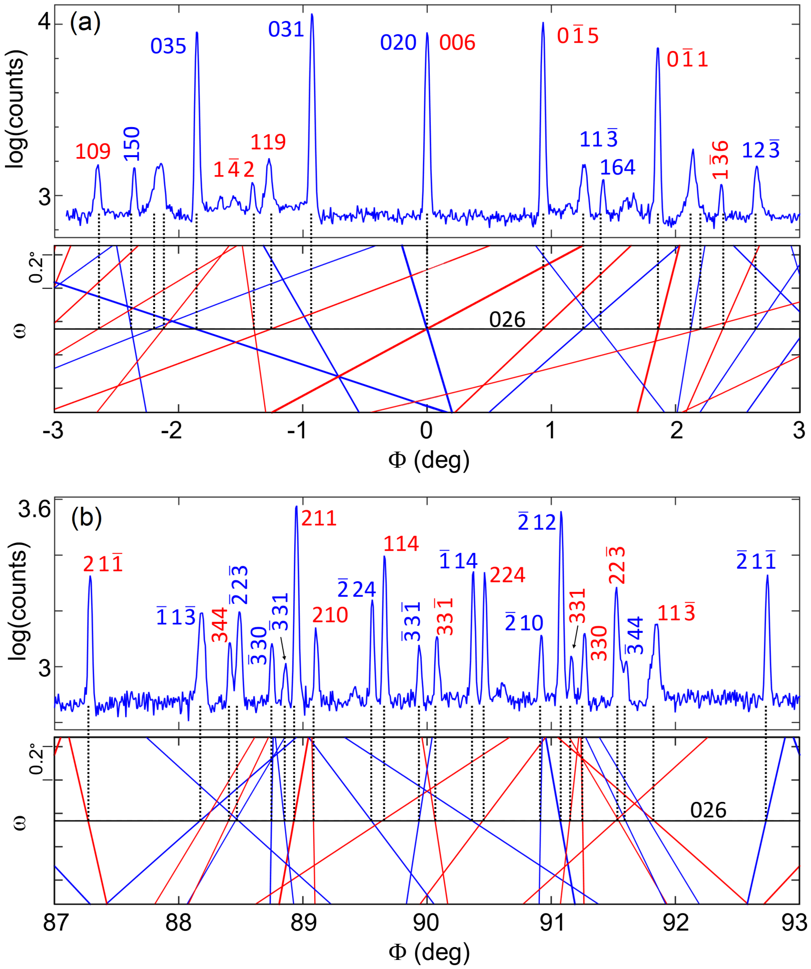

Fig. S1. ASN 026 Φ-scan around (a) $\Phi = 0$ and (b) $\Phi = 90°$ mirroring positions. Cu$K_{\alpha 1}$ radiation from a rotating anode generator. Rotation sense: clockwise with the 026 diffraction vector pointing to the observer. (Bottom panels of each scan) BC lines intercepting the 026 one (horizontal-black line) at the incidence angle ω as the 026 Bragg angle of 24.96°. Secondary reflection indices and BC lines in blue/red stand for the $g = -1/+1$ diffraction geometry.

S2. Asymmetry reading

Fig. S2 shows the uncertainties in asymmetry reading of MD peaks by line profile fitting with Gaussian function and sloping baseline, as computed by means of the bootstrapping statistical method. Dependence of the slope values with the used fitting range are reported in Fig. S3, as well as their relative error bars (inset) that remains nearly constant.

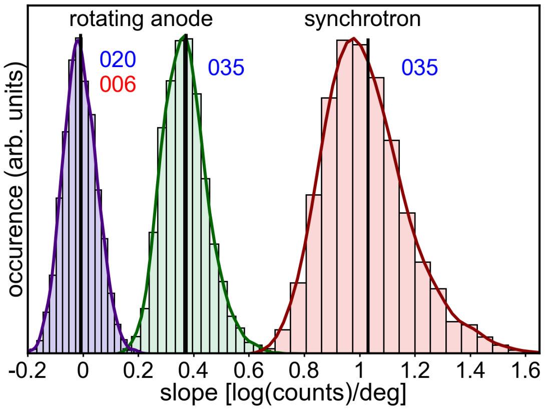

Fig. S2. Distribution of slope values for MD peaks 020/006 and 035, as measured with rotating anode (Fig.S1) and synchrotron (Fig. 7c, main text) sources. Mean slope values are indicated (vertical solid lines).

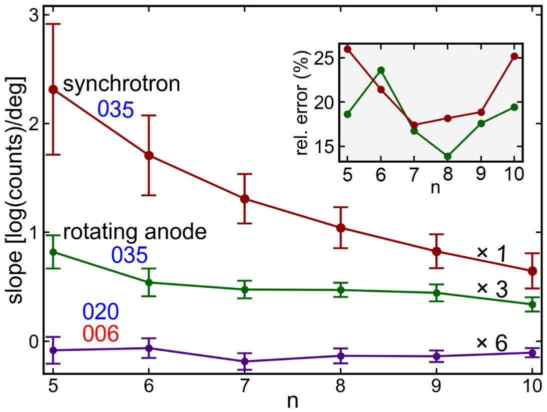

Fig. S3. Slope mean values for the MD peaks in Fig. S2 as a function of used fitting range of n times the respective peak FWHM. The relative error bars are given in the inset. Numbers on the right side are scale factors considered for better visualization.

S3. Triplet phases of ASN versus structural parameters

In the ASN 026 azimuthal scan, the measured asymmetry of each MD peak, with hkl indices, is related to the triplet phase $\Psi = \delta_H + \delta_{G-H} - \delta_G$ where $G = 026$, $H = hkl$, and δ_X is the structure factor phase of reflection X as in $F_X = |F_X| \exp(i\delta_X)$ (Penacchio, 2022; Morelhão, 2016). For the measured MDs, the calculated behaviour of Ψ as a function of model structure parameters x and z are shown in Fig. S4. When plotted against the phase δ_{026} of the reference reflection, it is clear that the behaviour of Ψ is dominated by the 026 reflection phase. As this phase is susceptible to both parameters, another dataset of profile asymmetries measured by using a different reference reflection is needed to better resolver the range of feasible values for these parameters. However, such experimental dataset is not available at the moment.

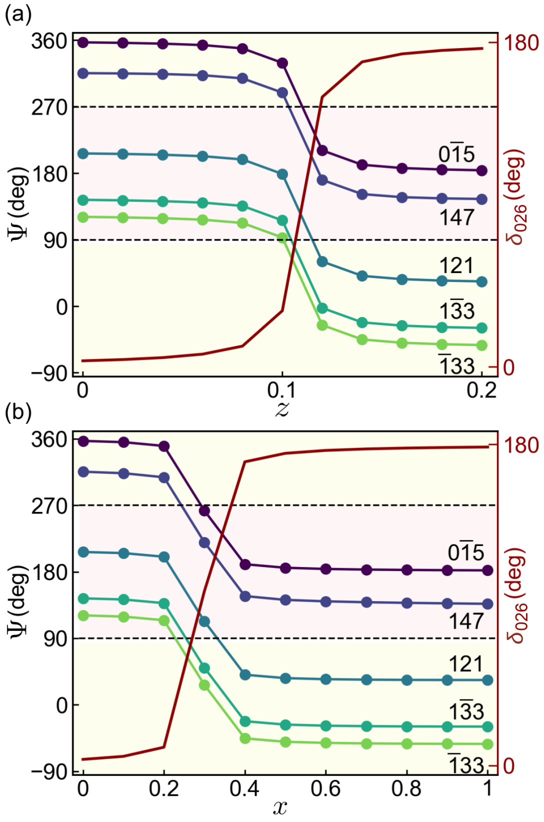

Fig. S4. Triplet phases Ψ (circles connected by colored lines) as a function of the ASN model structure parameters: (a) relative difference z in the rms displacement per atomic site with $x = 0$, and (b) ionic model x for the amino group with $z = 0$. As Ψ shifts across the 90° and 270° values (horizontal dashed lines), cos(Ψ) change signal and the corresponding peak asymmetry flips from HL to LH or vice-versa. Indices of secondary reflection H are indicated near each curve. 026 as the reference reflection G, whose values of its phase δ_{026} (solid line) as a function of parameters x and z are given on the vertical axis at the right.

References

Morelhão, S. L. (2016). *Computer Simulation Tools for X-ray Analysis*. Graduate Texts in Physics. Springer, Cham.

Penacchio, R. F. S., (2022). Pyddt - a helpful tool for planning x-ray dynamical diffraction experiments and analyzing ϕ-scans.
URL: *https://github.com/rafaela-felix/pyddt*
